# Groundwater depletion in the Middle East from GRACE with implications for transboundary water management in the Tigris-Euphrates-Western Iran region

**DOI:** 10.1002/wrcr.20078

**Published:** 2013-02-19

**Authors:** Katalyn A Voss, James S Famiglietti, MinHui Lo, Caroline Linage, Matthew Rodell, Sean C Swenson

**Affiliations:** 1 Science, Technology and International Affairs Program, School of Foreign Service, Georgetown UniversityWashington, District of Columbia, USA; 2 UC Center for Hydrologic Modeling, University of CaliforniaIrvine, California, USA; 3 Department of Earth System Science, University of CaliforniaIrvine, California, USA; 4 Department of Atmospheric Sciences, National Taiwan UniversityTaipei, Taiwan; 5 Hydrologic Sciences Branch, NASA Goddard Space Flight CenterGreenbelt, Maryland, USA; 6 Climate and Global Dynamics Division, National Center for Atmospheric ResearchBoulder, Colorado, USA

## Abstract

In this study, we use observations from the Gravity Recovery and Climate Experiment (GRACE) satellite mission to evaluate freshwater storage trends in the north-central Middle East, including portions of the Tigris and Euphrates River Basins and western Iran, from January 2003 to December 2009. GRACE data show an alarming rate of decrease in total water storage of approximately −27.2±0.6 mm yr^−1^ equivalent water height, equal to a volume of 143.6 km^3^ during the course of the study period. Additional remote-sensing information and output from land surface models were used to identify that groundwater losses are the major source of this trend. The approach used in this study provides an example of “best current capabilities” in regions like the Middle East, where data access can be severely limited. Results indicate that the region lost 17.3±2.1 mm yr^−1^ equivalent water height of groundwater during the study period, or 91.3±10.9 km^3^ in volume. Furthermore, results raise important issues regarding water use in transboundary river basins and aquifers, including the necessity of international water use treaties and resolving discrepancies in international water law, while amplifying the need for increased monitoring for core components of the water budget.

## 1. Introduction

Water scarcity in the Middle East, and the high frequency of conflict that emerges over what few resources do exist, is well established [e.g., *Amery and Wolf*, 2000; *Wolf and Newton*, 2007a; *Wolf*, 1998]. The recent drought that began in 2007 has further strained the limited water resources in the region [*Integrated Regional Information Networks*, 2010; *U.S. Department of Agriculture (USDA)*, 2008]. News reports detailed a dire situation in which fields lay fallow, wetland ecosystems were destroyed, and hundreds of farmers migrated to urban centers in search of employment [*Michel et al*., 2012; *Sullivan*, 2010]. Such drought typically amplifies the impact of management decisions by upstream users, as any decision to use or store water substantially influences total water availability within a river system, with potentially severe consequences for downstream users.

**Figure 1:**
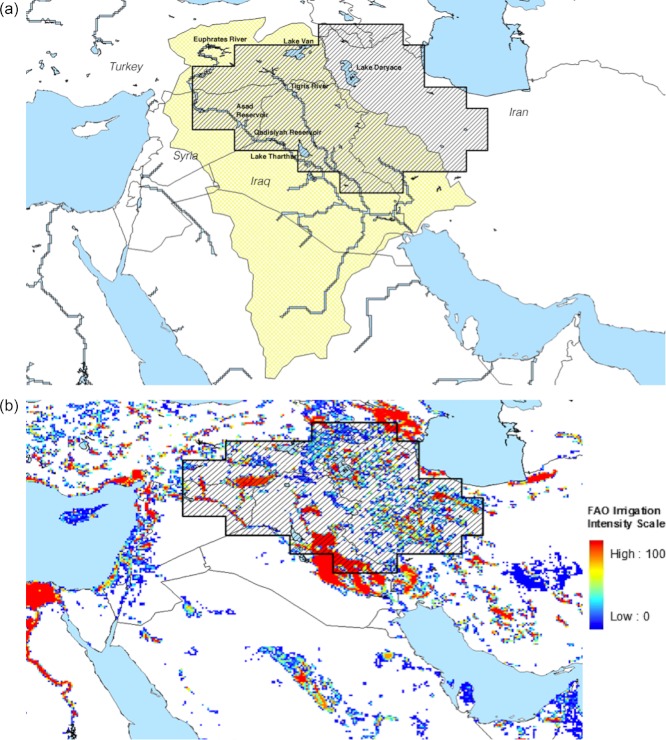
(a) Representation of selected study area. Thick black line with hashed fill represents the TEWI region for which GRACE data were extracted and supporting data sets compiled. All mass balance calculations were confined to this bounded region. Thin black lines represent political boundaries. Surface water bodies (light blue) were taken from the Global Lakes and Wetlands Database [*Lehner and Döll*, 2004]. Rivers are delineated in blue, and the respective watershed boundaries in crosshatched yellow [*Graham et al*., 1999]. (b) Small grid squares display percent of land under irrigation [*Siebert et al*., 2007]. Blue to red gradient represents intensity on a 0% to 100% scale, respectively.

Water management in the Tigris-Euphrates River Basin has been historically challenging [*Solomon*, 2010]. The Tigris-Euphrates is a transboundary river system (Figure 1a) that is shared among Turkey, Syria, Iraq, and, to a lesser extent, Iran. Both rivers contain extensive water management infrastructure, and the surface waters provided by the rivers are integral to the agricultural economies of the region [*Food and Agriculture Organization (FAO)*, 2009]. Struggles between the management decisions of the upstream user—Turkey—and the downstream demands of Syria and Iraq dominate the hydropolitics of the region [*Wolf and Newton*, 2007a]. In particular, the Southeastern Anatolian Project (Turkey's Greater Anatolia Project (GAP)) elevated tension among the three nations as Turkey acted unilaterally to construct over 20 dams on both the Tigris and Euphrates rivers [*Bayazit and Avci*, 1997]. This intensive infrastructure development has significantly altered the Tigris and Euphrates Basins in many ways. Turkish, Syrian, and Iraqi water managers now dictate the river flows with timed releases from the reservoirs. In addition, a complex system of transboundary groundwater aquifers underlies this region [*FAO*, 2009]. Domestic and international monitoring and regulation for the groundwater aquifers is lacking, despite the fact that it is a vital resource for the region, especially where and when surface water is unavailable.

Two major issues complicate water management in the region. First, there are no formal water allocation rights for both surface and groundwater. At the core of this dilemma are underlying differences in the interpretation of international water law [*United Nations*, 1997; *Weiss*, 2009], including its applicability to groundwater and to surface-groundwater interactions. These differences in interpretation severely limit the potential for any agreement for legal allocations or management policies for the Tigris and Euphrates Rivers.

A second challenge is the paucity of hydrologic data for the region. Inconsistent monitoring combined with a lack of data transparency and accessibility is a problem that plagues water managers around the globe, and the Tigris-Euphrates region is no exception. Such data scarcity and inaccessibility result in an incomplete understanding of water availability and use in this area of the Middle East. Although there have been other studies in the region [*Chenoweth et al*., 2011; *Jones et al*., 2008], *Kavvas et al*. [2011] showed that publicly available observations of streamflow, precipitation, and evaporation data are sparse to nonexistent, and if available, data sets are often incomplete. Classified, government-controlled data do exist, but access to these data requires the permission and cooperation of the respective governments. Access to groundwater information is similarly constrained, with limited or no data related to water table height or annual groundwater extraction available publically. Consequently, despite its importance, there have been few basin-wide hydrological studies using observational data for the Tigris-Euphrates Basin in recent years.

Satellite observations of time-variable gravity from the Gravity Recovery and Climate Experiment (GRACE) satellite mission [*Tapley et al*., 2004] present a new and valuable tool to fill these gaps in data availability and water monitoring [*Lettenmaier and Famiglietti*, 2006; *Rodell et al*., 2009; *Tiwari et al*., 2009; *Famiglietti et al*., 2011b]. GRACE provides a record of variations in total terrestrial water storage (defined as all of the snow, surface water, soil moisture, and groundwater) across the globe [e.g., *Rodell and Famiglietti*, 1999; *Wahr et al*., 2004; *Ramillien et al*., 2004; *Syed et al*., 2008]. Recent studies have demonstrated that water storage changes can be inferred from the GRACE data with sufficient resolution and accuracy to benefit water management [*Yeh et al*., 2006; *Rodell et al*., 2007; *Ramillien et al*., 2008; *Zaitchik et al*., 2008]. For example, GRACE data have been used to estimate rates of groundwater depletion [*Rodell et al*., 2009; *Tiwari et al*., 2009; *Famiglietti et al*., 2011b], flood potential [*Reager and Famiglietti*, 2009], drought [*Andersen et al*., 2005; *Yirdaw et al*., 2008; *Leblanc et al*., 2009; *Agboma et al*., 2009; *Chen et al*., 2010], and reservoir storage changes [*Swenson and Wahr*, 2009; *Wang et al*., 2011].

In this study, we used 84 months of GRACE data (January 2003 to December 2009) to examine the behavior of water storage in the north-central region of the Middle East, an area that includes most of the Tigris and Euphrates River Basins and western Iran. Additional data sets, including precipitation, evapotranspiration, streamflow, reservoir levels, and soil moisture, were compiled to help characterize the causes of observed variations and emerging trends. As an area that is well known for water scarcity and tension over transboundary waters, the Tigris-Euphrates region offers a compelling example of the power of satellite observations to provide insight into critical water resource issues in regions where hydrological observations are otherwise difficult to obtain. *Wada et al*. [2010] and *Siebert and Döll* [2008] developed methods to quantify changes in water resources in areas with limited observational data by using global hydrological and water resources models to model surface water discharge as well as groundwater recharge and to estimate water consumption based on statistics on population, gross domestic product (GDP), and irrigated areas. These methods highlight potential options when observational data are limited, and in addition to the approach followed here, in our opinion, may well provide an example of ‘best current capabilities’ in regions like the Middle East, where data access can be severely limited.

## 2. Data and Methods

### 2.1. Description of Study Region

The specific study area within the Tigris-Euphrates region (see Figure 1) was selected based on the analysis of regional trends present in the global GRACE data set. This region, shown in Figure 1a, displays a strong negative trend in total water storage and has an area of 753,960 km^2^. It includes most of the Tigris River Basin, the upper and middle section of the Euphrates River Basin, and western Iran. Consequently, we refer to the masked region in Figure 1a as the TEWI region, or simply, the study region.

The TEWI region spans the countries of Turkey, Syria, Iraq, and western Iran. Portions of Georgia and Azerbaijan, as well as all of Armenia, are also included in the study region, as are several large surface water bodies, namely, Lake Daryace, Lake Van, Lake Tharthar, the Asad Reservoir, and the Qadisiyah Reservoir. In addition to the area's surface water, a complex groundwater system [*German Federal Institute for Geosciences and Natural Resources (BRG) and United Nations Educational, Scientific, and Cultural Organization (UNESCO)*, 2010] underlies the TEWI region. Both the surface and groundwater components of the study area are essential to understand the dynamics of the freshwater storage within the region.

Land use within the broader area surrounding the study region must also be considered. This broader area encompasses southeast Turkey, where most of the water management infrastructure—dams, reservoirs, and canals—of the GAP project is located. Additionally, there is extensive irrigation for agriculture in the study region and the broader surrounding [*Siebert et al*., 2007]. Figure 1b highlights these attributes and characteristics.

### 2.2. GRACE-Derived Total Water Storage Data

We used 84 months, from January 2003 to December 2009, of GRACE-derived variations in total terrestrial water storage computed at the Center for Space Research at the University of Texas at Austin [*Chambers*, 2006]. The GRACE data were processed following the methods of *Swenson and Wahr* [2002, 2009] in order to yield monthly anomalies in total water storage (with respect to the mean of the study period) in terms of equivalent water height (mm) for the region outlined in Figure 1a. This method requires filtering of the GRACE data to reduce noise [*Swenson and Wahr*, 2006] and restore the associated lost signal over the defined TEWI area by scaling the data in order to recover the mass change estimate for the region [*Velicogna and Wahr*, 2006]. A scale factor of 1.09 was required in this work. The trend in total water storage (mm yr^−1^) was computed after removal of the annual signal. Note that the impact of contributions of the Caspian Sea, which abuts the TEWI region to the northeast, to variations in total water storage, were determined to be minor, accounting for less than 1.5% of the TEWI trend in total water storage.

### 2.3. Global Land Data Assimilation System Output

An extensive search for hydrological data in the region confirmed that in situ observations were publicly unavailable during the time period of interest. Consequently, to better understand the water balance dynamics in the study region, we used output from the NASA Global Land Data Assimilation System (GLDAS) [*Rodell et al*., 2004a] for precipitation, evapotranspiration, streamflow, soil moisture, and snow water equivalent. GLDAS is land surface modeling system that integrates global, satellite-based observational data to drive advanced simulations for climate and hydrologic investigations. For example, precipitation data come from the NOAA Climate Prediction Center Merged Analysis of Precipitation [*Xie and Arkin*, 1997], and near-surface air temperature, specific humidity, wind, and pressure are reported from the Goddard Earth Observation System Data Assimilation System as well as the Global Data Assimilation System [*Derber et al*., 1991; *Pfaendtner et al*., 1995]. These observational data are used to force the specific land surface model and provide the most accurate output.

In this study, we used the results of three land surface models from GLDAS—VIC [*Liang et al*., 1994, 1996], Noah [*Chen et al*., 1996; *Koren et al*., 1999], and CLM2 [*Dai et al*., 2003]—to balance the bias of any single model. GLDAS forcing and model outputs represent a viable alternative to overcome data inaccessibility [*Kato et al*., 2007; *Koster et al*., 2004; *Syed et al*., 2008; *Zaitchik et al*., 2010] in regions like the Middle East and may well represent the best available supporting data sets in similar areas where data are scarce or inaccessible.

Observed precipitation and model outputs for evapotranspiration and streamflow were combined in the water balance


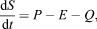
(1)

where 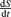 is change in water storage with time, *P* is precipitation, *E* is evapotranspiration, *Q* is streamflow, and all are expressed in mm mo^−1^. Model-derived 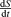 was compared with that determined from GRACE (discussed in section 3). Note that GRACE estimates of 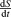 are the derivative, taken as the monthly difference (mm mo^−1^) of the anomalies of total water storage described above. GLDAS outputs for soil moisture and snow water equivalent were combined with satellite altimetry measurements of lake and reservoir heights (described below) in order to understand the respective contributions of each to the total water storage variations from GRACE. Outputs were prepared as monthly anomalies with respect to the study mean (mm) and as trends (mm yr^−1^) after removal of the annual signal.

### 2.4. Surface Water Altimetry Data

Remotely sensed altimetry data from the Hydroweb database at Laboratoire d'Etudes en Géophysique et Océanographie Spatiales (LEGOS) were used to calculate variations in water storage from surface water bodies [*Laboratoire d'Etudes en Géophysique et Océanographie Spatiales (LEGOS)*, 2011; *Crétaux et al*., 2011]. Complete data time series for five major surface water bodies—Lake Van, Lake Daryace (Lake Urmia), Lake Tharthar, the Asad Reservoir, and the Qadisiyah Reservoir—were available for the study period. These are among the biggest surface water bodies in the study area (by surface area) and account for approximately two-thirds of surface water in the study area, but do not include other large reservoirs, such as the Saksak, Ataturk, and Mossoul Reservoirs. Water level height data for these other reservoirs were unavailable for use in our study because the time series were incomplete.

Altimetry data for monthly water heights were converted to monthly changes in water volume using measurements of the mean surface area for the respective water bodies, as provided by LEGOS. We combined these values and divided by the total area of the region to calculate the anomaly of surface water storage (mm) with respect to the mean of the study period. The trend in surface water storage (mm yr^−1^) was also determined after the annual signal was removed. We used these data to ultimately infer the contribution of surface water variations to the total water storage variation observed by GRACE.

### 2.5. Estimating Groundwater Storage Changes

*Rodell and Famiglietti* [2002], *Yeh et al*. [2006], *Rodell et al*. [2007, 2009], *Strassburg et al*. [2007], *Tiwari et al*. [2009], *Famiglietti et al*. [2011b], and others have all demonstrated that the groundwater component of total water storage can be successfully isolated from the GRACE data. This approach, shown in equation (2), estimates monthly groundwater storage variations as the residual of the water storage balance, after subtracting the variations in snow water equivalent, surface water, and soil moisture storage from those of total water storage observed by GRACE. This is expressed as


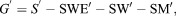
(2)

where *G* is groundwater storage, *S* is total water storage, SWE is snow water equivalent, SW is surface water storage, SM is soil moisture, and the primes indicate anomalies with respect to the mean of the particular component during the study period.

### 2.6. Error Analysis

Based on the errors of the other components, the trend error in GW was estimated using the following equation:


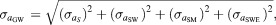
(3)

where 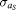 is the associated one-sigma trend error for total water storage from GRACE, 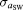 is the associated one-sigma trend error from the altimetry-derived surface water, 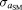 and 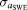 are the trend errors for GLDAS-computed soil moisture and snow water equivalent, respectively. The trend and trend error for each component of the water budget in the right-hand side of equation (2) are calculated after the annual signal is removed.


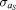
 and 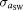 are computed by propagating the monthly error of *S*′ (11.3 mm) and SW′ (6.4 mm), respectively, onto the least-squares-estimated trends and are 0.6 mm yr^−1^ and 0.4 mm yr^−1^, respectively. Errors for soil moisture and snow water equivalent trends are the standard deviations of the trends computed from the three land models used in the GLDAS simulations (VIC, Noah, and CLM2), using the methods based on the study by *Kato et al*. [2007], which showed that the standard deviation among models was generally as large or larger than the difference between any one model (or the mean) and observed SM. This leads to errors of 1.9 mm yr^−1^ and 0.5 mm yr^−1^, respectively.

Monthly error on *G*′ is computed similarly to equation (3) and plotted as a gray shaded area in Figure 4. Monthly error on *S*′ is 11.3 mm and includes measurement and leakage errors. Monthly error on SW′ is 6.4 mm and is the mean of the combined five lakes' monthly errors. Monthly errors on SM′ and SWE′ are the standard deviation of the monthly SM′ and SWE′ computed from the three land surface models (VIC, Noah, and CLM2).

## 3. Results

### 3.1. Comparison of GRACE-Observed and GLDAS-Simulated Total Water Storage

**Figure 2:**
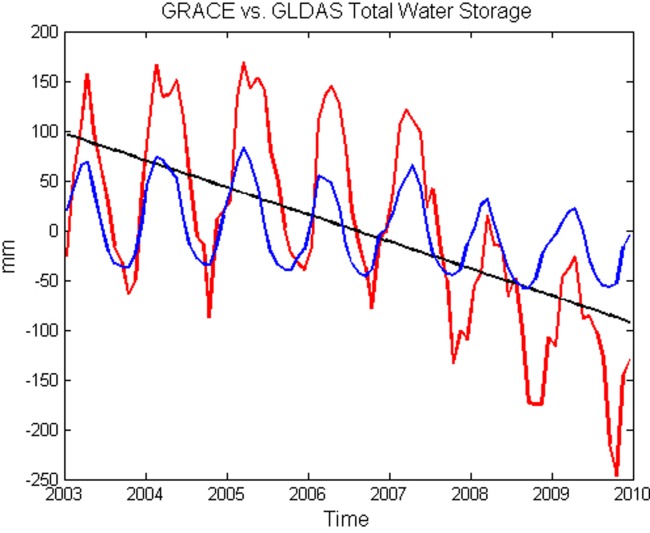
Total water storage anomaly (combined precipitation, evapotranspiration, and streamflow time series) produced by GLDAS when compared with GRACE total water storage anomaly for the TEWI region in Figure 1, from January 2003 to December 2009. The blue line is GLDAS total water storage, the red line is GRACE total water storage, and the black line is the GRACE total water storage trend (−27.2±0.6 mm yr^−1^). The associated error for GLDAS total water storage is the mean monthly standard deviation from the three land surface models used (Vic, Noah, and CLM2), whereas the GRACE total water storage monthly error is 11.3 mm for every month, which is the sum error from scaling and leakage.

GRACE observations of monthly terrestrial water storage anomalies were compared with the GLDAS-simulated anomalies for the study region, which were taken as the mean of the three land surface models (Figure 2). The comparison highlights three key issues that warrant further discussion.

First, numerous studies have demonstrated that the GRACE data capture natural water storage variations very well when compared with observations [*Rodell and Famiglietti*, 2001; *Rodell et al*., 2004a; *Rodell et al*., 2004b; *Syed et al*., 2008; *Swenson and Wahr*, 2006; *Swenson et al*., 2006; *Yeh et al*., 2006; *Syed et al*., 2009]. Second, however, the amplitude of the seasonal variations in GRACE-observed total water storage is generally greater than that simulated models, a result that is apparent in Figure 2. Several previous GRACE studies [*Wahr et al*., 2004; *Swenson and Wahr*, 2006; *Swenson and Milly*, 2006; *Niu et al*., 2006; *Syed et al*., 2008] point to missing or poor model representations of snow, surface water bodies and reservoirs, complete soil depth, and groundwater storage as the key reason why models cannot reproduce GRACE-observed storage amplitudes, as is the case here. *Niu et al*. [2006] found that adding a groundwater component to the CLM resulted in better simulations of the seasonal cycle of total water storage relative to GRACE, whereas *Lo et al*. [2010] used GRACE data to calibrate and improve model simulations of total water storage after adding a groundwater component.

Third, land surface model simulations of total water storage will deviate significantly from GRACE observations in areas where human water use and management (e.g., significant surface and groundwater storage and extraction) dominate the regional storage balance. For example, *Tiwari et al*. [2009] subtracted GLDAS total water storage simulations from GRACE data to isolate the human water management component of water storage change (primarily from groundwater pumping) in India. Figure 2 clearly shows that the GLDAS simulations cannot capture the human response to the drought, which begins after 2007. Although the models can simulate the natural variations due to decreasing precipitation (as evidenced by the generally-decreasing peaks shown by the blue line in Figure 2), because they do not parameterize surface and groundwater reservoir storage and extraction, irrigation, and other human uses of water, they are incapable of representing the heightened water withdrawals that occur during periods of drought [*Famiglietti et al*., 2011b]. Consequently, the models are unable to capture the decreasing water storage trend observed by GRACE. In fact, the difference between GRACE-observed and GLDAS-simulated total water storage trends is an indirect measure of surface and groundwater depletion during the drought period. In the rest of this section, we use the auxiliary observations and model data sets described in section 2 to better understand how the snow, surface water, soil moisture, and groundwater components behave during the time period studied, including human-driven rates of groundwater depletion.

Given the fact that the Tigris-Euphrates River Basin has extensive water infrastructure, the impacts of water management may significantly influence the trend in water storage anomalies in the TEWI region [*Swenson and Wahr*, 2009; *Wang et al*., 2011]. In the past, when GRACE data are compared with observational data, the water storage variations from GRACE matches quite well with observed trends [e.g., *Famiglietti et al*., 2011b; *Rodell et al*., 2007; *Syed et al*., 2008*; Yeh et al*., 2006]; therefore, we feel confident in assuming that GRACE-derived estimates of terrestrial water storage variations are accurate.

### 3.2. Total Water Storage

**Figure 3:**
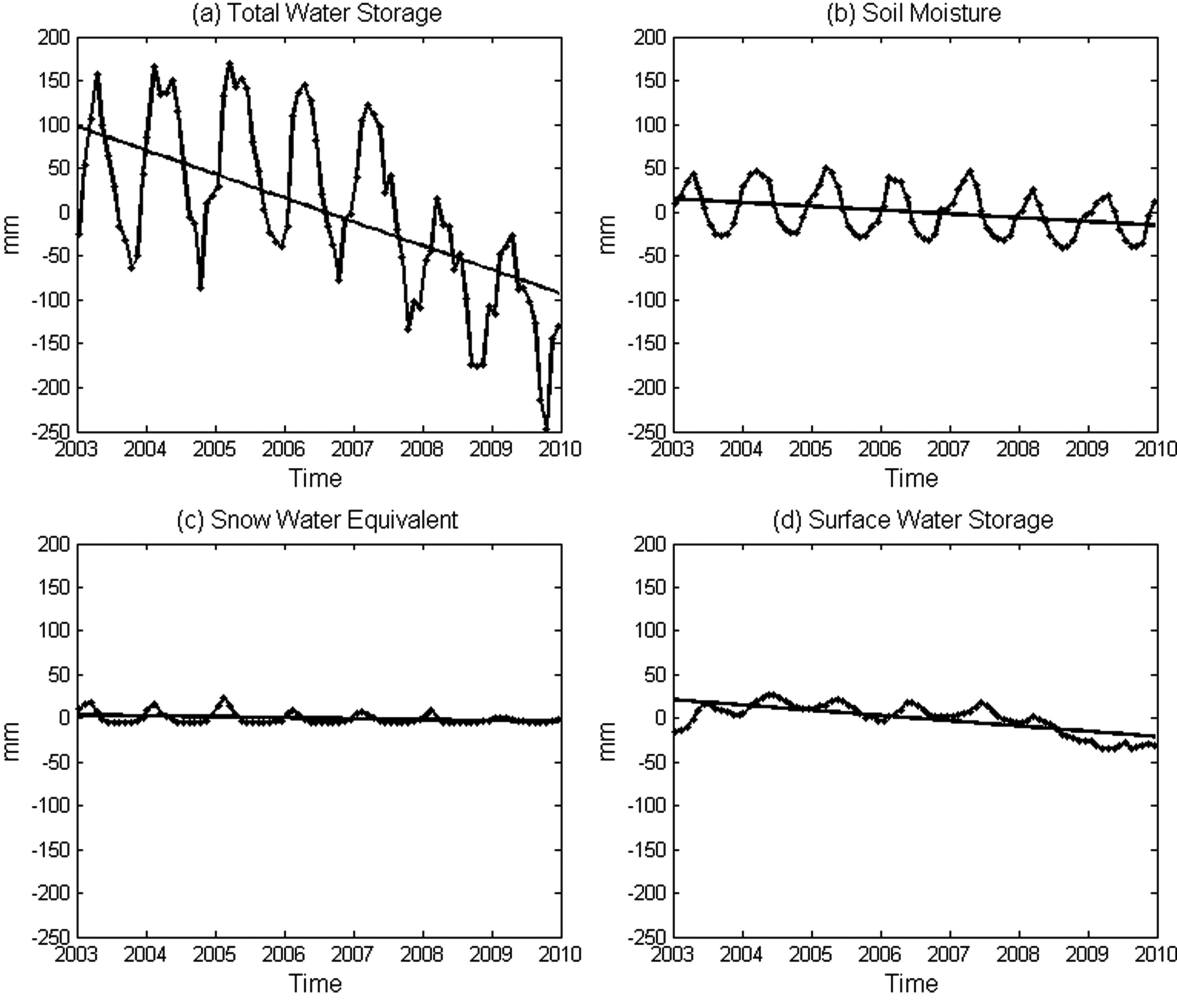
(a) Monthly total water storage anomalies and trend from GRACE for the study region from January 2003 to December 2009. The GRACE TWS trend is −27.2±0.6 mm yr^−1^. (b) Monthly soil moisture storage changes and trend from GLDAS. Soil moisture trend is −3.1±1.9 mm yr^−1^. (c) Monthly altimetry-based estimates of surface water storage changes and trend. Surface water storage trend is −5.9±0.4 mm yr^−1^. (d) Monthly snow water equivalent storage changes and trend from GLDAS. Snow water equivalent storage trend is −0.9±0.5 mm yr^−1^.

Figure 3a shows that from January 2003 to December 2009, the trend in GRACE-derived total water storage was −27.2±0.6 mm yr^−1^ for the study region. This equates to a −20.5±0.4 km^3^ volume loss of water each year, for a total volume loss of approximately −143.6±2.8 km^3^ during the study period. This rate of water loss is among the largest liquid freshwater losses on the continents [*Rodell et al*., 2009]. Water storage in the region shows a clear decline in the GRACE data, especially after 2007, which coincides with the beginning of a regional drought and subsequent changes in water use and availability. The 143.6±2.8 km^3^ loss during the 7-year study period is nearly equivalent in volume to the entire Dead Sea, which has an average volume of 147 km^3^.

**Figure 4:**
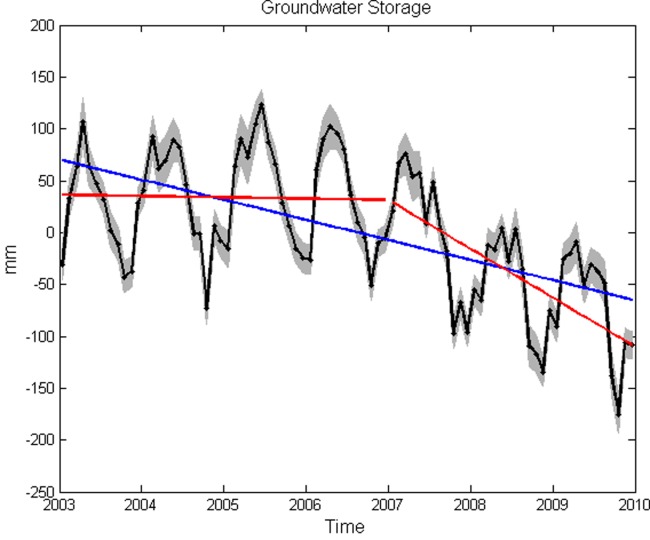
Monthly groundwater storage variations (as anomalies) and trend for the study region, from January 2003 to December 2009. Gray shaded area represents error, which is the monthly one-sigma error from the combined GRACE TWS, GLDAS-derived SM and SWE, and SW altimetry errors. Groundwater storage variations are shown by the black line. The blue line is the overall trend for the study period, which is −17.3±2.1 mm yr^−1^. The red lines represent the piecewise trends from January 2003 to December 2006 and January 2007 to December 2009, which are 4.9±3.1 mm yr^−1^ and −34.0±4.5 mm yr^−1^, respectively.

### 3.3. Snow Water Equivalent and Soil Moisture

Figure 3b shows the GLDAS output for soil moisture, and Figure 3c shows that for snow water equivalent. The trend in soil moisture was calculated as −3.1±1.9 mm yr^−1^, and the trend in snow water equivalent was −0.9±0.5 mm yr^−1^, representing volumetric losses of −2.3±1.4 km^3^ yr^−1^ and −0.7±0.4 km^3^ yr^−1^, respectively. These negative trends are largely climate driven and reflect the regional drought mentioned above. Together they account for roughly one fifth of the observed water losses.

### 3.4. Surface Water

Surface water storage anomalies are shown in Figure 3d. The trend for the study period was −5.9±0.4 mm yr^−1^ with an equivalent volume loss of −4.4±0.3 km^3^ yr^−1^, or nearly 20% of the total volume of water lost during the 84 month period studied. It is likely that our surface water trend underestimates the actual trend as only five water bodies comprised the calculation, Lakes Daryace, Van, and Tharthar and the Asad and Qadisiyah Reservoirs, and changes in the respective surface areas are not taken into account. As mentioned above, the five water bodies for which altimetry data were available likely represent approximately two-thirds of the actual surface water storage changes within the boundaries of the study area (based on mapped area of all surface water bodies). By assuming a constant surface area, our estimate of monthly surface water storage is overestimated; however, with this assumption of constant surface area, the rate of decrease, or trend, is actually underestimated. Thus, in both cases (missing reservoirs and neglecting surface area shrinkage), we underestimate the surface water storage trend. Consequently, our estimates in the residual groundwater storage component may be overestimated. As discussed above, we acknowledge the shortcomings and additional uncertainties that this contributes to the study; however, we suggest that this work offers an example of a “best available” approach for regions where in situ data are inaccessible.

### 3.5. Groundwater

Following equation (2), groundwater storage anomalies were calculated as the residual after subtracting the snow water equivalent, surface water, and soil moisture components of the water budget from the anomalies in total water storage observed by GRACE. Figure 4 shows the monthly groundwater storage anomalies estimated in this manner. Both the seasonal cycle and the clear decline of groundwater storage after 2007 are apparent in Figure 4. The trend in groundwater was −17.3±2.1 mm yr^−1^ (−13.0±1.6 km^3^ yr^−1^; −91.3±10.9 km^3^ for the entire study period), a considerable loss that accounts for 63% of the total water storage change from 2003 to 2009. As seen in Figure 4, the declining trend in groundwater coincides with the 2007 drought. The trends in the water storage component are summarized in Table 1, as well as in total water storage, discussed in this section.

**Table 1:** Water Storage Trends in the TEWI Region From 2003 to 2009

Water Storage Component	Trend (mm yr^−1^)	Volume Lost (km^3^ yr^−1^)	Total Volume (km^3^)
GRACE total water storage	−27.2±0.6	−20.5±0.4	−143.6±2.8
Surface water	−5.9±0.4	−4.4±0.3	−31.1±2.1
Soil moisture	−3.1±1.9	−2.3±1.4	−16.3±10.0
Snow water equivalent	−0.9±0.5	−0.7±0.4	−4.9±2.5
Groundwater (GRACE-SW-SM-SWE)	−17.3±2.1	−13.0±1.6	−91.3±10.9
Groundwater from 2003 to 2006	4.9±3.1	3.7±2.3	14.7±9.3
Groundwater from 2007 to 2009	−34.0±4.5	−25.6±3.4	−76.9±10.1

## 4. Discussion

### 4.1. Hydrologic Trends in the Tigris-Euphrates-Western Iran region

The GRACE data indicate a total water volume loss of nearly 144 km^3^ over the 7-year period studied. This loss is particularly alarming for regions such as the TEWI region, which is already facing severe water scarcity. The analyses presented here suggest that groundwater depletion is the largest single contributor to the observed negative trend, accounting for approximately 60% of the total volume of water lost, the majority of which occurred after the onset of drought in 2007. As we do not have in situ data to validate these conclusions, we acknowledge that there is uncertainty in this analysis, which we have attempted to quantify. However, we believe that this combination of remotely sensed and modeled data provides a very valuable alternative for understanding hydrologic changes occurring in data-scarce regions.

Although in situ hydrologic observations are nearly impossible to obtain, the results presented here are consistent with published reports from the region. Land subsidence due to overabstraction of groundwater near Tehran, Iran, is well documented [*Alipour et al*., 2008; *Lashkaripour et al*., 2005; *Motagh et al*., 2008]. A Brookings Institution report highlighted the displacement of hundreds of thousands of people from northern Iraq due to lack of water [*Michel et al*., 2012]. Our study complements these reports by providing a holistic, regional assessment of water losses in the region, while quantifying key storage changes such as those of groundwater and surface waters.

**Figure 5:**
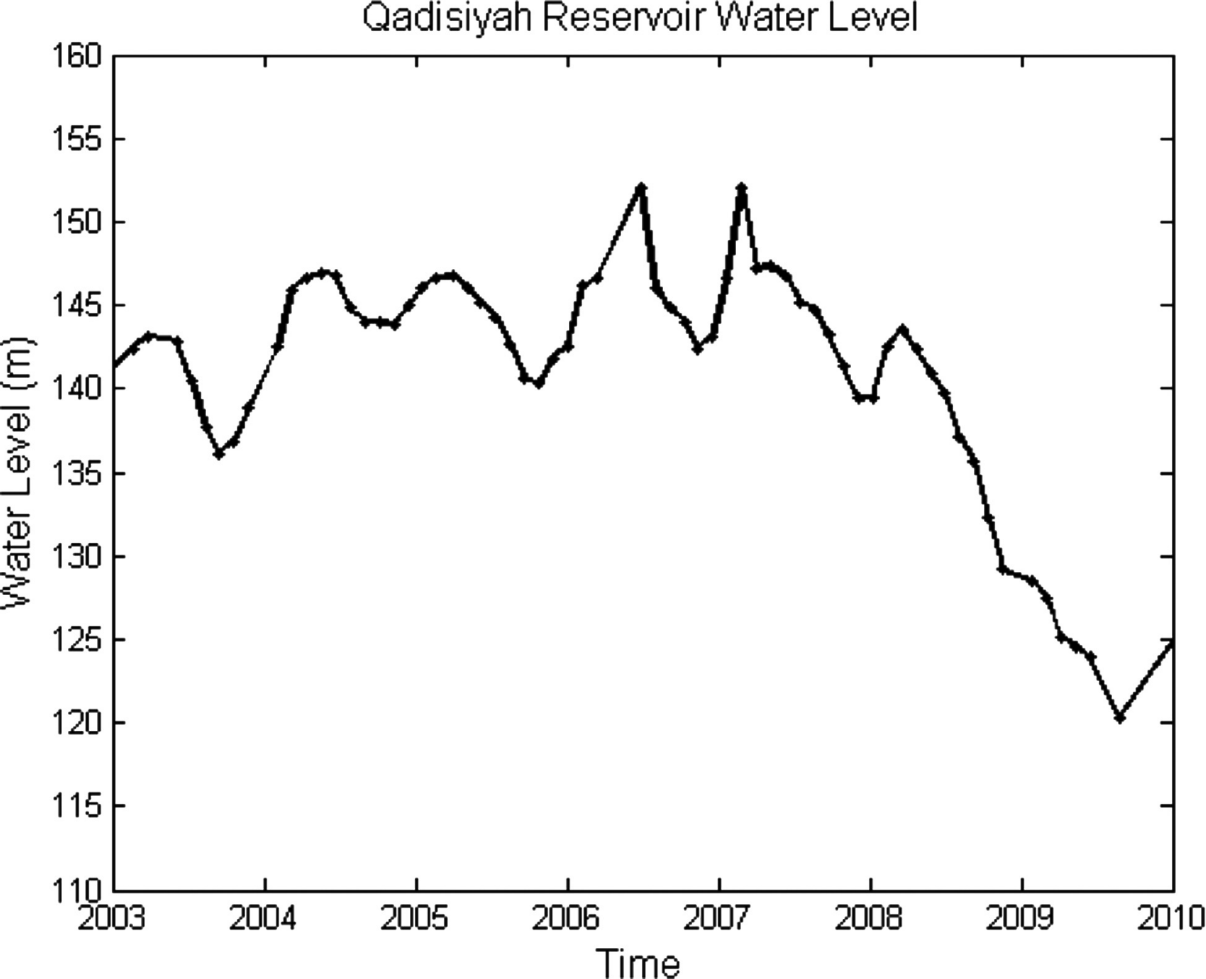
Storage variations for the Qadisiyah Reservoir in Iraq. From altimetry data for January 2003 to December 2009 [*LEGOS*, 2011].

Water use behavior in Iraq during the study period followed the typical pattern of increased groundwater abstraction in response to drought and declining surface water availability [*Famiglietti et al*., 2011b]. Based on the altimetry data, the level of Iraq's major reservoir on the Euphrates River, the Qadisiyah Reservoir, sharply declined in 2007 (see Figure 5). According to *Chulov* [2009], by the end of 2007, Euphrates River stream flow had decreased to approximately 70% of its normal flow by the time it crossed into Iraq. Without water from reservoir storage or river flow to replenish it, Iraq had little choice but to increase their reliance on groundwater. *Chulov* [2009] noted that the Iraqi government dug approximately 1000 new groundwater wells from 2007 to 2009, while abstracting 80% of the country's groundwater reserves in response to the decline in surface water resources. These 1000 new wells are referred only to those constructed by the Iraqi government. It is highly likely that civilians constructed numerous additional private wells to meet their agricultural and domestic needs. This rapid increase in groundwater consumption, with no replenishment from precipitation or streamflow, is an important driver of the groundwater losses that are estimated in this study.

### 4.2. Transboundary Water Management Implications

The water management response to the drought in the Tigris-Euphrates Basin portion of the study area, and the overall negative TEWI water storage trend, raises the critical issue of effective, science-informed transboundary water management strategies. Unfortunately, international water law fails to provide a guiding principle for transboundary management in the Tigris-Euphrates Basin as its application varies from nation to nation. Consequently, Turkey, Syria, and Iraq have foregone any legally binding water allocations or management practices for the two rivers. In the absence of binding agreements, downstream users—Syria and Iraq—are dependent on upstream water management decisions—by Turkey—which ultimately determine the downstream flows of the Tigris and Euphrates [*Salman*, 2004; *UN Environmental Program (UNEP)*, 2008; *Wolf and Newton*, 2007b].

Without an effective international water management strategy, each country is free to act unilaterally. Economically, each is dependent on Tigris and Euphrates water for agricultural irrigation, a core component of the national economies. In fact, the main purpose of the expansive GAP was to provide water resources for agriculture expansion in southeast Turkey [*Directorate of State Hydrolic Works (DSI)*, 2009; *Harris*, 2009; *Ozdogan et al*., 2006], and Syria, Iraq, and Iran use much of their limited water resources to support the goals of the agriculture sector [*FAO*, 2009]. As the final downstream user of the Tigris and Euphrates Rivers, Iraq receives only the streamflow that remains after appropriations and diversions by Turkey and Syria [*Zawahri*, 2006].

The consequences of this lack of transboundary management are clear from this study. The ultimate downstream user is left with little surface water availability and must deplete its nonrenewable reserves of groundwater. After the drought began in 2007, agricultural productivity declined for all three nations. Upstream, Turkey was least affected, with most crop yields slightly declining or remaining constant. However, downstream in Syria and Iraq, significant, larger declines occurred in all crops, particularly barley [*USDA*, 2011]. The decline in agricultural output significantly influences economic stability in the region and will continue to be a threat owing to perennial limitations on water availability and the emerging threats of climate change, including more prolonged drought.

### 4.3. New Tools for Collaborative Water Management

Our analysis highlights the role that the GRACE mission, and other recent and near future advances in hydrologic remote sensing, can play as important new tools for regional and transboundary environmental decision making. The study described here offers a valuable and unique opportunity to understand hydrologic trends in a data-inaccessible region such as the region of the Middle East studied here. Using GRACE data, we reported that nearly 144 km^3^ of water was lost in the TEWI region from 2003 to 2009. Using supplementary data sets from global hydrologic models and from satellite altimetry in a mass balance framework, we determined that the drivers of this trend were a combination of drought and corresponding increases in groundwater use. Approximately 91 km^3^ of the total amount of water lost during the study period came from groundwater.

Our analysis also placed this research in its regional economic and political context. Economically, this crisis resulted in the loss of agricultural yields, unemployment, emotional hardship, and mass migrations. Politically, the response to the drought represents a missed opportunity for cooperative transboundary water management.

Hopefully, we provided valuable insights for a region with little transparency in its water management decisions and few publicly available data sources. Although there is no substitute for ground-based observational data, GRACE and other emerging satellite water sensors will provide a unique tool for water management across the globe. Presented with a holistic picture of changing water availability, while confronted with the common problems of preserving and protecting this shared resource, nations and states may experience new incentives to collaborate on water management issues across political boundaries. Moreover, the synoptic view from space may ultimately render data denial and management opacity policies obsolete, as water management practices are being increasingly revealed from space [*Swenson and Wahr*, 2009; *Tiwari et al*., 2009; *Rodell et al*., 2009; *Famiglietti et al*., 2011b], and simultaneously, they are being better represented in regional and global hydrological models [*Famiglietti et al*., 2011a; *Wood et al*., 2011].

Emerging advances in hydrologic remote sensing and hydrological models, combined with enhanced access to observational data, suggest that the opportunity to construct the most accurate and holistic picture of freshwater availability, for a particular region or across the globe, is now on us. Such science-informed studies are essential for more effective, sustainable, and in transboundary regions, collaborative water management.
